# Corazonin Neurons Contribute to Dimorphic Ethanol Sedation Sensitivity in *Drosophila melanogaster*

**DOI:** 10.3389/fncir.2022.702901

**Published:** 2022-06-22

**Authors:** Adeola Oyeyinka, Mehul Kansal, Sean M. O’Sullivan, Claudia Gualtieri, Zachary M. Smith, Fernando J. Vonhoff

**Affiliations:** Department of Biological Sciences, University of Maryland Baltimore County, Baltimore, MD, United States

**Keywords:** invertebrate, neurobiology, neuroethology, sex-reversal, sexual-dimorphism, insects, neuromodulation

## Abstract

Exposure to alcohol has multiple effects on nervous system function, and organisms have evolved mechanisms to optimally respond to the presence of ethanol. Sex differences in ethanol-induced behaviors have been observed in several organisms, ranging from humans to invertebrates. However, the molecular mechanisms underlying the dimorphic regulation of ethanol-induced behaviors remain incompletely understood. Here, we observed sex differences in ethanol sedation sensitivity in *Drosophila* Genome Reference Panel (DGRP) lines of *Drosophila melanogaster* compared to the absence of dimorphism in standard laboratory wildtype and control lines. However, in dose response experiments, we were able to unmask dimorphic responses for the control mutant line *w*^1118^ by lowering the testing ethanol concentration. Notably, feminization of the small population of Corazonin (Crz) neurons in males was sufficient to induce female-like sedation sensitivity. We also tested the role of the transcription factor *apontic* (*apt*) based on its known expression in Crz neurons and its regulation of sedation responses. Interestingly, loss of function *apt* mutations increased sedation times in both males and females as compared to controls. No significant difference between male and female *apt* mutants was observed, suggesting a possible role of *apt* in the regulation of dimorphic ethanol-induced responses. Thus, our results shed light into the mechanisms regulating sex-differences in ethanol-induced behaviors at the cellular and molecular level, suggesting that the genetic sex in a small neuronal population plays an important role in modulating sex differences in behavioral responses to ethanol.

## Introduction

Exposure to ethanol has significant effects on neuronal and brain function in humans and animals. Ethanol effects range from stimulating at acute and low concentrations, to depressant at chronic and high concentrations, involving the possible development of alcohol use disorders (AUDs) (Pohorecky, 1991). The literature surrounding the effects of ethanol in different organisms have shown that the molecular and neuronal basis underlying ethanol responses are evolutionary conserved, including in invertebrates such as *Caenorhabditis elegans* (Pandey et al., 2021), honey bees (Mustard et al., 2019), and *Drosophila* fruit flies (reviewed in Scholz and Mustard, 2013; Lathen et al., 2020). Fruit flies have been a useful animal model to study responses to ethanol, not only because they encounter ethanol in fermented food under natural conditions, but also because of the high level of conservation in behavioral responses to ethanol (hyperactivity, tolerance, and sedation) as well as in the molecular mechanisms identified in both mammals and flies (Grotewiel and Bettinger, 2015; Park et al., 2017).

Whereas early studies in flies focused on genes involved in ethanol metabolism such as alcohol dehydrogenase (ADH) (Cavener, 1979) and aldehyde dehydrogenase (ALDH) (Heinstra et al., 1989), recent studies have diversified their molecular candidates, including molecules involved in memory formation and the reward system, such as the neurotransmitters dopamine (Bainton et al., 2000; Scaplen et al., 2020) and octopamine (Scholz et al., 2000; Scholz, 2005), as well as the neuropeptides NPF (Shohat-Ophir et al., 2012) and Corazonin (Crz) (McClure and Heberlein, 2013; Sha et al., 2014; Varga et al., 2016). In fact, Crz neurons have been the focus of recent studies involving male flies and ethanol-dependent behaviors. This is in part because activation of Crz neurons in males causes ejaculation (Tayler et al., 2012; Zer-Krispil et al., 2018), which is described to be rewarding and to reduce motivation to consume ethanol (Zer-Krispil et al., 2018). By contrast, silencing of Crz neurons increases ethanol sedation times (McClure and Heberlein, 2013), making Crz-dependent signaling a key player in the male reward and stress mechanism (Khan et al., 2021; Zandawala et al., 2021).

Interestingly, although sex-dependent responses to ethanol have been observed in humans (Miller et al., 2009; Ceylan-Isik et al., 2010) and flies (Devineni and Heberlein, 2012), most studies include either females or males, and only a few include both. Clear dimorphic differences in ethanol-induced pharmacokinetics and behaviors have been previously reported in flies, with males showing increased hyperactivity and longer sedation times as compared to females (Devineni and Heberlein, 2012). However, the molecular mechanisms underlying the dimorphic regulation of ethanol responses remain poorly understood. At the molecular level, some of the observed sex differences in ethanol responses have been linked to the female-specific splicing factor Transformer (Tra), which regulates splicing of the neural sex determination gene *fruitless* (*fru*). *fru* encodes a male-specific group of putative transcription factors called FruM (Demir and Dickson, 2005; Kimura et al., 2005; Stockinger et al., 2005). Although sex determining genetic factors are required pan-neuronally, including the ∼1500 *fru*-expressing neurons to regulate dimorphic ethanol responses, it remains unclear whether genetic sex plays a role in a smaller subset of neurons to determine dimorphism in ethanol-induced behaviors. Furthermore, the Myb/SANT-containing transcription factor *apontic* (*apt*) is known to be required in Crz neurons for ethanol sedation sensitivity in males, but its role in ethanol-induced differences between males and females remains unexplored (McClure and Heberlein, 2013).

Here, we investigate the molecular and cellular mechanisms underlying sexually dimorphic responses in ethanol sedation sensitivity. We used an established assay to test sedation sensitivity by exposing male and female flies to a high ethanol vapor concentration (65%–73%) (Sandhu et al., 2015). We show that dimorphism in ethanol sedation sensitivity is observed in four different lines from the *Drosophila* Genome Reference Panel (DGRP), whereas classic wildtype (Canton S and Oregon R) and control (*w*^1118^) lines show no dimorphic differences. A dimorphic sedation response could be observed in *w*^1118^ flies by lowering the ethanol concentration as indicated by our dose response experiments. Additionally, sex-reversing experiments demonstrate that feminization of Crz neurons in males is sufficient to reduce sedation time to female-like levels. Lastly, we observe no sex differences in ethanol sedation in *apt* mutant flies, suggesting a potential role of *apt*-dependent transcription in dimorphic ethanol-induced behaviors.

## Materials and Methods

### Fly Stocks and Maintenance

*Drosophila* Genome Reference Panel lines: DGRP-774 (RRID:BDSC_25205), DGRP-73 (RRID:BDSC_28131), DGRP-304 (RRID:BDSC_25177), and DGRP-229 (RRID:BDSC_29653), as well as CRZ-GAL4 (RRID:BDSC_51976), and UAS-Tra (RRID:BDSC_4590) lines were obtained from the Bloomington Drosophila Stock Center. The *apt* mutant lines (Gellon et al., 1997; Starz-Gaiano et al., 2008) as well as Oregon R, Canton S, and *w*^1118^ (RRID:BDSC_3605) were obtained from the Starz-Gaiano Lab at UMBC. DGRP lines are fully sequenced inbred lines derived from a natural population from Raleigh, NC, United States (Mackay et al., 2012). *w*^1118^ is a strain of flies that have a specific mutation in the *white* gene, which can potentially exist in any genetic background. Flies were reared at 25°C in a 12/12 h light/dark incubator on standard plastic vials. Our food recipe included 9900 ml of water, 800 ml of molasses, 652 g of cornmeal, 130 g of yeast, 34 g of agar, 57.6 ml propionic acid, and 13.4 g methyl paraben. Males from fly lines carrying the two *apt* mutant alleles tested here, *apt*^*KG*05830^ and *apt*^167^ were crossed to a fly stock that carried the X chromosome from the DGRP-774 line and visible markers on the second chromosome (+^*DGRP*774^; CyO/Sp). F1 CyO males (+^*DGRP*774^/>; *apt*/CyO) were backcrossed to the maker line (+^*DGRP*774^; CyO/Sp), and subsequently, F2 males and females were selected against Sp to make stable fly lines (+^*DGRP*774^; *apt*^*KG*05830^/CyO and +^*DGRP*774^; *apt*^167^/CyO) that were maintained and expanded in the lab. We used resources provided by flybase.org, which serves as an essential database for our studies (Larkin et al., 2021).

### Ethanol Sedation Assays

Behavioral assays were performed following established sedation protocols (Sandhu et al., 2015), with minor modifications. Briefly, 10 2-day to 4-day-old non-virgin females and males of each genotype were subjected to brief (less than 5 min) carbon dioxide anesthesia no less than 24 h before behavioral testing. Flies were transferred into clean polystyrene tubes (22.5 mm × 90 mm) and plugged using two Bonded Dense Weave Cellulose Acetate closures. The top of the first plug lined up with a line 2 cm down from the top of each tube. Flies were allowed to acclimate to room temperature (23°C) for 30 min. One milliliter of the testing ethanol concentration was added to the plug. Then, a second plug was added as low as 1 cm down from the top of each tube, creating a 1 cm wide vaporization chamber (Chan et al., 2014). Tubes were gently tapped every 5 min and the loss-of-righting reflex (LORR) was quantified (number of flies unable to right themselves) (van der Linde and Lyons, 2011) for a maximum of 60 min. Sedation time 50 (ST50) values represent the time required for 50% of flies to show LORR and were calculated using linear extrapolation.

### Statistical Analysis

Graphs and statistical analysis were performed using GraphPad Prism 9.3.1. Normality was tested using the Shapiro–Wilk test. Statistical significance was established using the parametric one-way ANOVA and Šidák or Holm-Šídák’s corrections for multiple comparisons. Data is presented as scatter plots showing the observed individual values along with descriptive statistics (mean and SEM). In all graphs, statistical significance is shown by ^****^p < 0.0001, ^***^p < 0.001, ^**^p < 0.01, and *p < 0.05.

### Quantitative Reverse Transcription PCR

Control and experimental flies were separated by sex, snap frozen in an ethanol dry ice slush and homogenized in DNA/RNA Shield (Zymo Research). Total RNA was extracted using Quick-RNA MiniPrep (Zymo Research) according to manufacturer’s instructions and stored at −20°C. Total RNA was reverse transcribed using iScript cDNA Synthesis Kit (Bio-Rad) according to manufacturer’s instructions. A Bio-Rad CFX96 Real-time Detection System was used for amplification (Saadin and Starz-Gaiano, 2018; Le et al., 2021). The sequence for primers targeting *apt* were obtained from Wang et al. (2020) and were normalized to endogenous *actin42A* transcript levels with primer pair sequences from Ponton et al. (2011). Experiments were performed on three biological replicates, each in technical triplicates using groups of three 3–7 days old flies for each genotype. The data were presented as fold change as compared to DGRP-774 males. Primers used for *apt* were: Forward, GGGAACGCACCCAGAACTG, Reverse, GACCGGCATCCAATCTCTTGT. Primers for *actin42A* were: Forward, GCGTCGGTCAATTCAATCTT, Reverse, AAGCTGCAACCTCTTCGTCA. Statistical analysis was performed by one-way ANOVA followed by Dunnett’s multiple comparisons test using GraphPad Prism 9.3.1.

## Results

**FIGURE 1:**
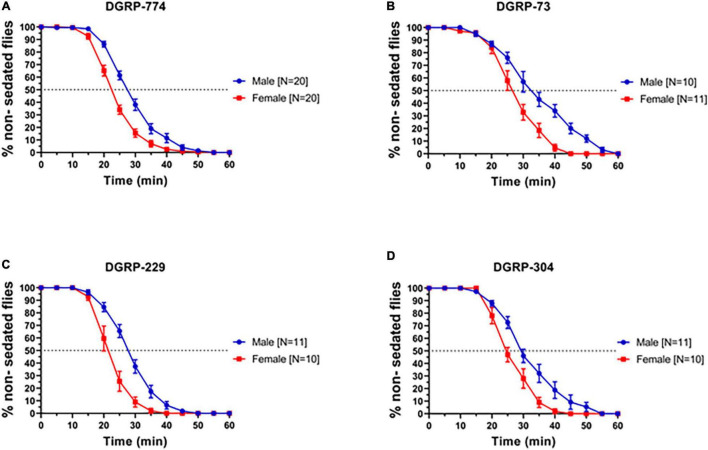
*Drosophila* Genome Reference Panel lines show dimorphic responses to ethanol sedation. Sedation curves of males and females exposed to 73% ethanol vapor for fly lines DGRP-774 **(A)**, DGRP-73 **(B)**, DGRP-229 **(C)**, and DGRP-304 **(D)**. Sample size (*N*) indicates the number of runs. Each run included a total of 10 flies per single run.

**FIGURE 2:**
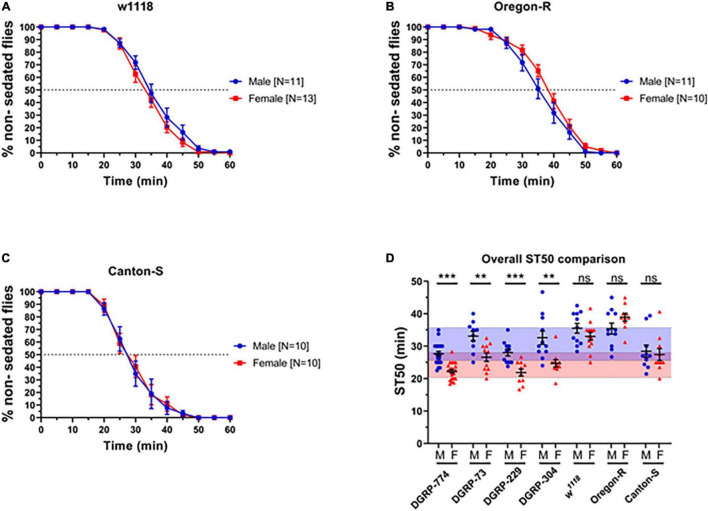
Classic control lines show no dimorphic responses to ethanol sedation when exposed to 73% ethanol vapor. Sedation curves of males and females for fly lines *w*^1118^ **(A)**, Oregon R **(B)**, and Canton-S **(C)**. ST50 values of DGRP and control lines show that DGRP lines show dimorphic ethanol responses at 73%, whereas classic laboratory-adapted fly lines show no sex differences **(D)**. Red-shaded area represents the total female average ST50 from all DGRP runs (24.16 ± 3.86 min). Blue-shaded area represents the total male average ST50 male (30.63 ± 5.08 min). Statistical significance was tested by one-way ANOVA with Šidák corrections and is shown by ***p < 0.001 and **p < 0.01. ns, not significant.

In order to examine whether we can observe dimorphic responses to ethanol in adult flies, we first tested a range of wildtype lines for their sedation response following previously established protocols using a LORR assay (Sandhu et al., 2015). Based on previous studies, we exposed flies to a concentration of 73% ethanol and observed sigmoidal sedation kinetics that indicated dimorphic behavior in four lines from the DGRP: DGRP-774, -73, -304, and -229 (Mackay et al., 2012) previously used in degeneration studies (Ederer et al., 2018; Gabrawy et al., 2019, 2022). A well-established method to measure sedation response or sensitivity is to quantify the time required for 50% of flies to sedate (ST50) (McClure and Heberlein, 2013; Sandhu et al., 2015). In all four DGRP lines, males sedated more slowly than females indicated by a higher ST50 value (Figure 1). For example, DGRP-774 males had an ST50 of 27.65 ± 0.76 min (*N* = 20 runs, 10 flies in each run; mean ± SEM; see section “Materials and Methods”) whereas females had an ST50 of 22.28 ± 0.60 min (*N* = 20 runs; *p* = 0.0003, ANOVA) (Figures 1A, 2D). The total male average ST50 from all DGRP male trials was 30.63 ± 5.08 min (Figure 2D, blue-shaded area), whereas the total female average ST50 was calculated as 24.16 ± 3.86 min (Figure 2D, red-shaded area).

We next sought to determine the effect of genetic background by testing additional lines that have served as common control lines in fly research for much longer than the DGRP lines. These include the wildtype lines Canton-S and Oregon-R, as well as the white mutant *w*^1118^. Surprisingly, we did not observe dimorphic sedation rates using 73% ethanol in any of the classical control lines (Figure 2). For example, *w*^1118^ males had an ST50 of 35.51 ± 1.5 min (*N* = 11), while *w*^1118^ females had an ST50 of 33.01 ± 1.2 min (*N* = 13; *p* = 0.20) (Figure 2A). We hypothesized that the used ethanol concentration (73%) may be too high to dissect apart differences between male and female sedation responses. Therefore, we next performed a dose response assay on DGRP-774 and *w*^1118^ to test whether discernible effects of ethanol on dimorphism could be observed.

**FIGURE 3:**
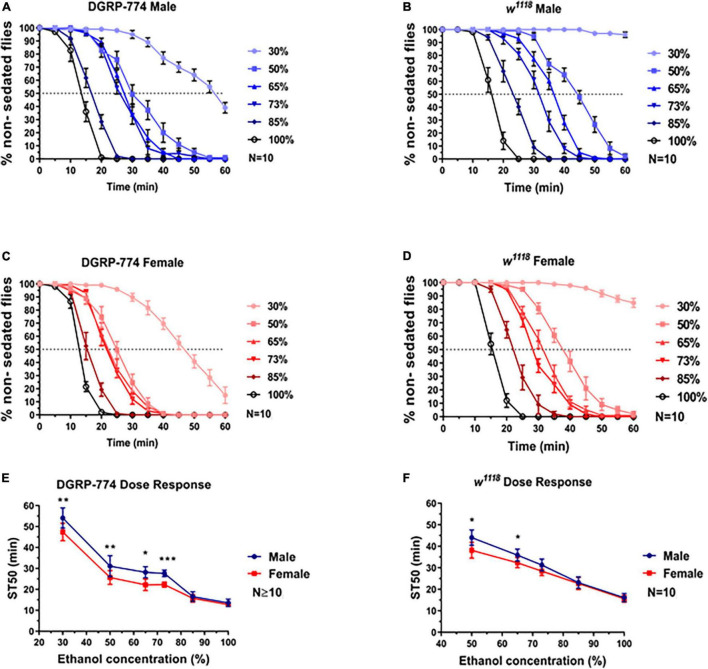
Dose required to observe sexual dimorphism varies by genetic background. While the DGRP-774 showed dimorphism at concentrations ≤73% **(A,C,E)**, *w*^1118^ was not dimorphic until concentrations ≤65% **(B,D,F)**. Statistical significance was tested by one-way ANOVA with Šidák corrections and is shown by ***p < 0.001, **p < 0.01, and *p < 0.05.

Indeed, we observed a dimorphic sedation response in *w*^1118^ at lower ethanol concentrations than DGRP-774 (Figure 3). While DGRP-774 showed significant differences between males and females in sedation rates at concentrations between 30% and 73% (Figure 3E), *w*^1118^ only showed significance at 50% and 65% (Figure 3F). The effect sizes with confidence interval as a function of ethanol percentage for ST50 values of DGRP-774 and *w*^1118^ flies were further analyzed to compare the strength of the observed dimorphic effects (Supplementary Figure 1). An ST50 was not determined for 30% as *w*^1118^ flies did not begin to sedate until ∼1 h after ethanol exposure. Thus, our data indicate that dimorphic differences can be masked at higher ethanol concentrations dependent on the genetic background.

Next, we sought to further understand dimorphic responses to ethanol at the cellular and molecular level. Previous studies used sex-reversal experiments to elegantly demonstrate that masculinized females showed male-like responses to ethanol such as increased ST50 values as compared to control females (Devineni and Heberlein, 2012). Consistently, it was also shown that feminized males presented female-like responses. However, these experiments were based on the manipulation of the female-specific splicing factor Tra and Transformer 2 (Tra2) in all neurons or in a large population of neurons, such as in the cells expressing the neuronal sex determination gene *fru*. Therefore, we asked whether sex-reversing a small number of cells would be sufficient to regulate the observed sex-dependent responses in our ethanol sedation assay. Neurons expressing the neuropeptide Crz represent a good candidate to regulate dimorphic ethanol responses, as silencing of Crz neurons as well as Crz RNAi-knockdown have been previously shown to reduce ethanol sedation sensitivity (McClure and Heberlein, 2013). Adult brains contain 6-8 Crz neurons on each hemisphere (Nässel and Zandawala, 2020; Khan et al., 2021), representing a smaller set than the *fru*-expressing population.

**FIGURE 4:**
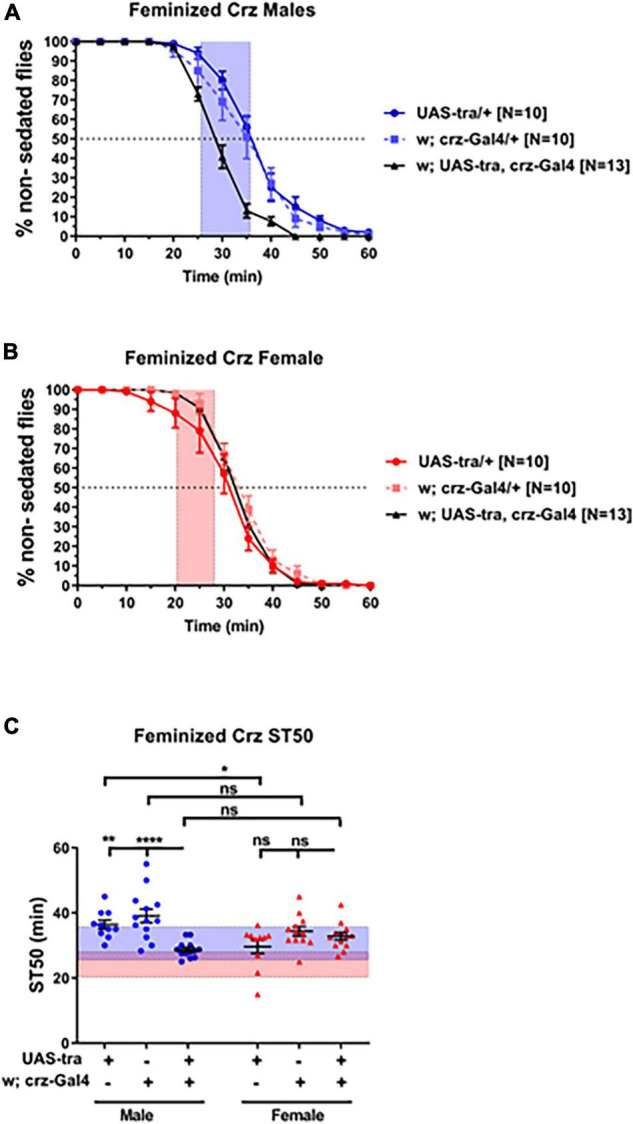
Feminization of Crz neurons reduces male sedation time. Expression of the feminizing factor UAS-Tra in Crz neurons using the Crz-GAL4 driver reduced sedation times in males **(A)** to female-like levels **(B)** when tested using 73% ethanol. Heterozygous animals were obtained from crosses between *w*^1118^ flies and either the GAL4 or the UAS line. For reference, red-shaded area in all panels represents the total female average ST50 at 73% from all DGRP runs (24.16 ± 3.86 min). Blue-shaded area represents the total male average ST50 male (30.63 ± 5.08 min). Statistical significance in ST50 values **(C)** was tested by one-way ANOVA with Šidák corrections and is shown by ****p < 0.0001, **p < 0.01, and *p < 0.05. ns, not significant.

In order to investigate whether sex-determining genetic factors function autonomously within Crz neurons to regulate ethanol sedation, we expressed the UAS-Tra construct via Crz-GAL4 to feminize Crz neurons. Males carrying both the UAS and the GAL4 constructs showed a significant reduction in sedation time compared to males from each control line (Figures 4A,C; *p* < 0.0001; ANOVA). Male heterozygous control animals with hybrid backgrounds showed higher ST50 values than females (UAS-Tra/+, 36.42 vs. 29.62 min, *p* = 0.0455; and Crz-GAL4/+, 39.09 vs. 34.35 min, *p* = 0.2223), whereas heterozygous males with feminized Crz neurons showed lower ST50 values than females from the same cross (UAS-Tra/crz-GAL4; 28.67 vs. 32.80 min, respectively). These results suggest that the sex determination pathway may function in a cell autonomous manner to modulate ethanol sedation sensitivity and that dimorphic ethanol responses can be regulated by the genetic sex of a small subpopulation of neurons.

**FIGURE 5:**
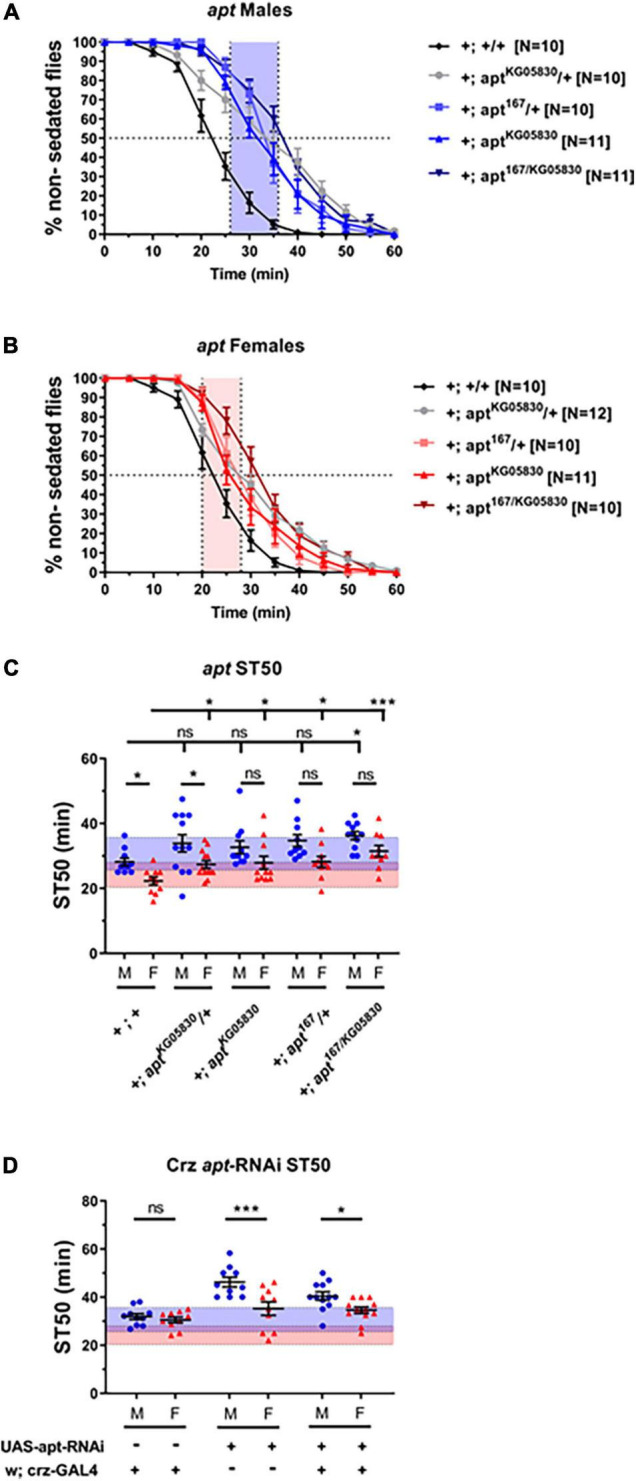
Flies bearing *apt* mutations do not show sexually dimorphic sedation responses. Flies bearing loss-of-function mutations in *apt* were tested using 65% ethanol show higher sedation times than DGRP-774 control flies in males **(A)** and females **(B)**. Significant differences between male and female ST50 values are observed in DGRP-774 control flies and *apt*^*KG*05830/+^ heterozygotes, whereas no dimorphism was observed between *apt*^167/KG05830^ heteroallelic mutants, *apt*^*KG*05830^ homozygotes, and *apt*^167/+^ heterozygotes males and females **(C)**. All *apt* mutants shown in panel **(C)** were crossed to generate stable lines that have the X chromosomal background of the DGRP-774 line (see section “Materials and Methods”). Heterozygous animals were obtained by crossing females from these stable *apt* lines with DGRP-774 males. Expression of an *apt*-RNAi construct *via* Crz-GAL4 did not disrupt sexually dimorphic sedation responses **(D)**. For reference, the red-shaded and the blue-shaded area represents the female and male average ST50 at 73% as described in Figure 4. Statistical significance was tested by one-way ANOVA with Šidák corrections and is shown by ***p < 0.001 and *p < 0.05. ns, not significant.

We next sought to investigate the molecular mechanisms regulating dimorphic Crz function in ethanol sedation sensitivity. Although several genes have been identified to regulate ethanol sedation responses, few have been shown to function specifically in identified neurons. One such gene is the transcription factor *apt*, which has been shown to function in Crz neurons to promote sedation in males (McClure and Heberlein, 2013). However, whether *apt* function regulates ethanol sedation in females remains an unexplored question. Therefore, we tested the dimorphic effects of loss of *apt* on ethanol sedation sensitivity by using two *apt* loss-of-function alleles, which were crossed into the DGRP-774 genetic background (see section “Materials and Methods”). The hypomorphic allele *apt*^*KG*05830^ contains a P-element insertion 5′ to the start site of the *apt*-RA transcript (Bellen et al., 2004), whereas *apt*^167^ contains a point mutation in the DNA binding domain (Gellon et al., 1997) and is a lethal null allele. By crossing both lines, we obtained viable *apt*^167/KG05830^ heteroallelic mutants (Starz-Gaiano et al., 2008). Our results show that DGRP-774 control flies and *apt*^*KG*05830/+^ heterozygotes showed significant dimorphic responses, whereas no significant differences between male and female ST50 responses were observed in the other *apt* mutant alleles (Figure 5). Additional crosses were set to assess the effect of different genetic backgrounds on these *apt* flies, which included crosses to obtain heterozygous flies for the *w*^1118^ background or the CyO balancer on the second chromosome. We observed consistent trends in all cases, where *apt*^*KG*05830/+^ heterozygotes showed significant dimorphic responses, but no significant differences were observed in different *apt*^167/+^ heterozygotes (Supplementary Figure 2).

Additionally, all *apt* mutant backgrounds showed slightly increased sedation times in males compared to DGRP-774 male controls, but a statistically significant difference was only observed in the *apt*^167/KG05830^ heteroallelic mutant males. By contrast, a significant difference in sedation times was observed for all *apt* mutant females tested when compared to DGRP-774 female controls (Figure 5C). These results suggest that *apt* functions in both males and females to promote dimorphic sedation responses, although an effect from the genetic background on the observed sedation responses in *apt* mutants cannot be fully ruled out. To better understand these results, we performed RT-qPCR analysis on *apt* transcript levels from whole animals in DGRP-774 controls and the different *apt* mutants. Our control data of *apt* expression was consistent with previously reported dimorphic differences in the Flyatlas2 dataset (Leader et al., 2018; Krause et al., 2021), showing a higher overall *apt* expression in males than in females (Supplementary Figure 2). Interestingly, the *apt*^*KG*05830^ allele led to a significant reduction of *apt* transcript levels in females, whereas it had a modest, more variable effect in decreasing *apt* levels in males. By contrast, male *apt*^167/+^ heterozygotes showed a significant decrease in *apt* levels, whereas female *apt*^167/+^ heterozygotes showed similar *apt* levels as female controls. These results suggest that the *apt*^*KG*05830^ allele affects females at the transcriptional level, while the effects from the *apt*^167/+^ allele may be at the functional level due to the mutated DNA binding domain.

Corazonin neurons have been previously described to express *apt* to regulate sedation responses in males (McClure and Heberlein, 2013). Therefore, we tested whether disruption of *apt*-dependent signaling exclusively in Crz neurons would be enough to phenocopy the disruption of dimorphic sedation responses we observed in *apt* mutants. Targeted *apt*-knockdown to Crz neurons did not affect the dimorphic differences in sedation responses as males expressing an *apt*-RNAi construct showed significantly higher ST50 values than females (40.45 vs. 34.61 min; *p* = 0.04, ANOVA; Figure 5D). These results suggest that *apt*-dependent signaling may be required in different parts of the nervous system beyond Crz neurons to regulate dimorphic sedation responses.

## Discussion

### Genetic Background as Strong Contributor to Sexual Dimorphism

Our results indicate that sexually dimorphic responses to ethanol are genotype-dependent similar to previous studies testing several wildtype strains (Devineni and Heberlein, 2012) as well as DGRP lines when examined in a genome-wide association study (GWAS) aiming to identify candidate genes associated with natural variation in alcohol sensitivity (Morozova et al., 2015). Intriguingly, we observed no dimorphic differences in classic control lines when tested under the same conditions as the more recently isolated DGRP lines. Differences in dimorphic ethanol responses between laboratory-adapted fly populations have been reported in previous studies of the ADH locus (Oakeshott et al., 1984; Barbancho et al., 1987), but the precise underlying mechanisms remain unknown. Additionally, whereas some studies observed no dimorphic responses in *w*^1118^ flies (Devineni and Heberlein, 2012; Sandhu et al., 2015), we were able to observe dimorphism in this line after performing dose response experiments (Chan et al., 2014). However, the contribution of the genetic background to sedation responses was noticeable, particularly when fly lines carrying different genetic constructs in the *w*^1118^ background showed distinct effects on dimorphic sedation sensitivity. When tested with 73% ethanol, no dimorphism was observed in *w*^1118^ homozygotes and w; Crz-GAL4/+ heterozygotes, but it was observed in w; UAS-Tra/+ heterozygotes. Besides the overall genetic background, an additional explanation for this discrepancy could involve effects caused by differences in the insertion site as well as expression of the two genetic constructs. In addition to the presence or lack of GAL4 expression in these flies, both Crz-GAL4 and UAS-Tra constructs include a *mini-white* gene that leads to a variable level of eye pigmentation (Silicheva et al., 2010). A differential expression of *mini-white* may modulate various responses in *w*^1118^ flies as previously described for male-male courtship (Hing and Carlson, 1996), ethanol sensitivity (Chan et al., 2014), copulation success (Xiao et al., 2017), and neuronal degeneration (Ferreiro et al., 2017). Interestingly, whereas flies heterozygous for these constructs tend to show higher ST50 values than the calculated baseline from wildtype and *w*^1118^ homozygotes, males with both constructs (w; Crz-GAL4/UAS-Tra) to induce feminization of Crz neurons showed the opposite effect, namely a strong reduction of ethanol sensitivity. Therefore, although strong effects from the genetic background cannot be ruled out, the most parsimonious explanation for the decreased ST50 observed in Crz-GAL4/UAS-Tra males may involve the feminization of Crz neurons in these animals.

### Masculinization of Corazonin Neurons

Given the effect that feminization of Crz neurons had on sedation times, future studies could examine whether masculinization of Crz neurons by UAS-tra2 RNAi expression (Devineni and Heberlein, 2012) would also be sufficient to delay sedation in females. Loss of Tra2 function causes females to produce male specific isoforms of Dsx and Fru thus masculinizing Crz neurons. Pan-neuronal masculinization produced weaker effects on sedation than feminization (Devineni and Heberlein, 2012), likely due to reduced RNAi penetrance or due to the presence of additional non-FruM neurons preventing complete neural masculinization. Additionally, future studies would focus on immunohistochemistry approaches or single-cell RNA-seq of masculinized and feminized Crz neurons, as evidence of Dsx or Fru expression in Crz neurons is still lacking. It is possible that a cascade of responsive genes downstream of Dsx or Fru would set up the differential responses in male and female brains, which ultimately control dimorphic ethanol-dependent behaviors. Due to the developmental role of these molecules, we expect Dsx and Fru to be expressed during larval or pupal stages at higher levels than during adulthood. Our results show how genetic sex regulates a specific subset of neurons in order to module alcohol-induced behaviors, thus providing insight into the molecular mechanisms underlying sexually dimorphic ethanol responses. Moreover, our results provide further evidence to support the observation that ethanol responses are strongly regulated by neuronal function, whereas physiological factors such as body weight have a weaker effect on ethanol-induced behaviors (data not shown; see also McClure et al., 2011).

### Conservation of Molecular Mechanisms

From an evolutionary perspective, it seems that a variety of mechanisms to regulate the animals’ exposure to ethanol as well as ethanol-dependent behaviors have evolved in the nervous system in different animals. Most genes involved in behavioral responses to ethanol show high degrees of conservation among species. For example, ADH is expressed in most living organisms (Holmes, 1994), and most genes associated with ethanol-related behavior in flies have orthologs in *C. elegans* (Grotewiel and Bettinger, 2015; Scholz, 2019). However, the marine animal *Trichoplax adhaerens*, which lacks muscles and a nervous system, expresses ADH and ALDH, but not *corazonin* or *apt*, as revealed by genomic and transcriptomic analyses (Srivastava et al., 2008; Wong et al., 2019). This might suggest that *corazonin* and *apt* evolved at a later time during the establishment of more complex ethanol-dependent behaviors than just ethanol degradation. In contrast to mechanisms well conserved among species, the dopamine/ecdysteroid receptor (DopEcR), a G-protein coupled receptor for dopamine and the insect steroid hormone ecdysone, regulates ethanol responses in flies (Petruccelli et al., 2016; Aranda et al., 2017), but lacks mammalian orthologs, despite sharing more protein sequence similarity to vertebrate β-adrenergic receptors than to the *Drosophila* family of dopaminergic receptors (Petruccelli et al., 2020a). These results suggest that alternative mechanisms to ethanol exposure may exist, which go beyond ethanol processing and degradation. Such evolving mechanisms and molecules may be adopted by neuronal networks to facilitate appropriate behavioral responses in a changing environment and influence processes driven by natural selection. An intriguing question would address the evolutionary processes that led to the establishment of sexually dimorphic behaviors. Future studies using additional DGRP lines can be performed to map additional genes that contribute to dimorphism in the context of ethanol sedation sensitivity (Morozova et al., 2015; Engel et al., 2019; Petruccelli et al., 2020b). Furthermore, elucidating molecular and cellular mechanisms of sexual dimorphism may be relevant in understanding fundamental differences in not only brain function but also gender-dependent neurological diseases such as autism (Retico et al., 2016; Walsh et al., 2021) or Alzheimer’s disease (Podcasy and Epperson, 2016; Rahman et al., 2019) and their study in animal models, including rodents (Schäfer et al., 2007; Grabrucker et al., 2016) and flies (Bellosta and Soldano, 2019; Mariano et al., 2020; Jalali et al., 2021).

### Dimorphic *Apontic*-Dependent Regulation

The *apt*^*KG*05830^ allele is located at the start site of the *apt*-RA transcript (Bellen et al., 2004), which was the only transcript previously described to be expressed in male brains (McClure and Heberlein, 2013). Recent tissue specific expression data in Flyatlas2 (Leader et al., 2018; Krause et al., 2021) confirms that *apt*-RA is the only transcript expressed in both male and female brains at comparable levels. Our RT-qPCR data shows differences in the way that each allele affects *apt* expression, showing the *apt*^*KG*05830^ allele a strong suppression of *apt* transcripts in females, whereas the *apt*^167^ allele showed a significantly decreased expression in males. These results suggest that *apt* expression may be regulated in a dimorphic manner and that *apt* mutations may have a different effect in males and females. Despite such sex-dependent differences in the regulation of *apt*, our results indicate that LOF mutations in *apt* affects both males and females, disrupting sexually dimorphic sedation responses. However, targeted *apt*-knockdown to Crz neurons in males and females did not disrupt dimorphism, suggesting that *apt*-dependent signaling is required in a wide range of populations of neurons that regulate dimorphic behaviors. Some potential candidates may include neurons involved in the reward system (e.g., NPF and octopamine) as well as Fru-expressing neurons as described above. Furthermore, future studies would contrast *apt*-dependent regulation of gene transcription in males and females during development and adulthood to potentially unravel different roles as previously described for Crz (McClure and Heberlein, 2013). Together these experiments will shed light into the molecular mechanisms underlying gene transcription within specific populations of neurons in a dimorphic manner to generate a proper behavioral response to alcohol exposure.

## Data Availability Statement

The raw data supporting the conclusions of this article will be made available by the authors, without undue reservation.

## Author Contributions

AO, MK, and SO’S were involved in performing sedation experiments. ZS, CG, and FV performed the molecular biology techniques and some sedation experiments. All authors were involved in collecting and analyzing data, contributed to the manuscript drafting and revising, and approved the submitted version.

## Author Disclaimer

Any opinions, findings and conclusions or recommendations expressed in this material are those of the author(s) and do not necessarily reflect the views of the National Science Foundation (NSF).

## Conflict of Interest

The authors declare that the research was conducted in the absence of any commercial or financial relationships that could be construed as a potential conflict of interest.

## Publisher’s Note

All claims expressed in this article are solely those of the authors and do not necessarily represent those of their affiliated organizations, or those of the publisher, the editors and the reviewers. Any product that may be evaluated in this article, or claim that may be made by its manufacturer, is not guaranteed or endorsed by the publisher.

## Supplementary Material

Supplementary Figure 1 Estimation graphics of ST50 values from DGRP-774 **(A)** and *w*^1118^ **(B)** males and females. The mean difference for five ethanol concentrations (50, 65, 73, 85, and 100%) is shown in the Cumming estimation plots using the web application available at https://www.estimationstats.com (Ho et al., 2019). The raw data is plotted on the upper axes; each mean difference is plotted on the lower axes as a bootstrap sampling distribution. Mean differences are depicted as dots; 95% confidence intervals are indicated by the ends of the vertical error bars. The effect sizes and CIs are reported above as: effect size (CI width: lower bound; upper bound). A total of 5000 bootstrap samples were taken; the confidence interval is bias-corrected and accelerated. The *p*-value(s) reported are the likelihood(s) of observing the effect size(s) if the null hypothesis of zero difference is true. For each permutation *p*-value, 5000 reshuffles of the control and test labels were performed.

Supplementary Figure 2 **(A)** Heterozygous *apt* mutant flies show similar trends in sedation responses between males and females in different genetic backgrounds. Animals heterozygous for the *apt*^*KG*05830^ allele and either for the *w*^1118^ background or the CyO balancer show sexually dimorphic sedation responses similar to *apt*^*KG*05830/+^ flies heterozygous for the DGRP-774 background as shown in Figure 5C. For the *apt*^167^ allele, the progeny from the same cross (between females from the *apt*^167^/CyO stable line and DGRP-774 males) was compared. Whereas no significant dimorphic responses were observed in *apt*^167/+^ heterozygotes, dimorphism was observed between female and male CyO/+ siblings. Statistical significance was tested by one-way ANOVA with Šidák corrections and is shown by ^***^*p* < 0.001, ^**^*p* < 0.01, and **p* < 0.05. **(B)** *apt* and *actin42A* transcript levels were measured by qPCR from replicates using 3–7 days old flies. Fold change expression is normalized to DGRP-774 males. Significance values are shown for comparisons in-sex to DGRP-774 flies and between sexes within each genotype.

Supplementary Table 1 Details of the statistical analyses conducted in this study.
