# Supplementary figures and images for: Corazonin Neurons Contribute to Dimorphic Ethanol Sedation Sensitivity in Drosophila melanogaster
Source: Front Neural Circuits. 2022 Jun 22;16:702901. doi: 10.3389/fncir.2022.702901 (PMC9256964; doi:10.3389/fncir.2022.702901)

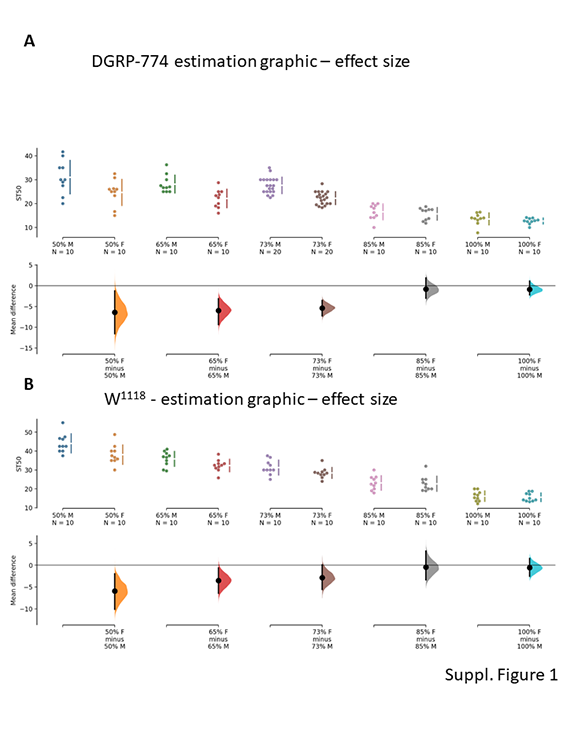

Supplement: Supplementary Figure 1 — Estimation graphics of ST50 values from DGRP-774 (A) and w1118 (B) males and females. The mean difference for five ethanol concentrations (50, 65, 73, 85, and 100%) is shown in the Cumming estimation plots using the web application available at https://www.estimationstats.com (Ho et al., 2019). The raw data is plotted on the upper axes; each mean difference is plotted on the lower axes as a bootstrap sampling distribution. Mean differences are depicted as dots; 95% confidence intervals are indicated by the ends of the vertical error bars. The effect sizes and CIs are reported above as: effect size (CI width: lower bound; upper bound). A total of 5000 bootstrap samples were taken; the confidence interval is bias-corrected and accelerated. The p-value(s) reported are the likelihood(s) of observing the effect size(s) if the null hypothesis of zero difference is true. For each permutation p-value, 5000 reshuffles of the control and test labels were performed. [file Image_1.tif]

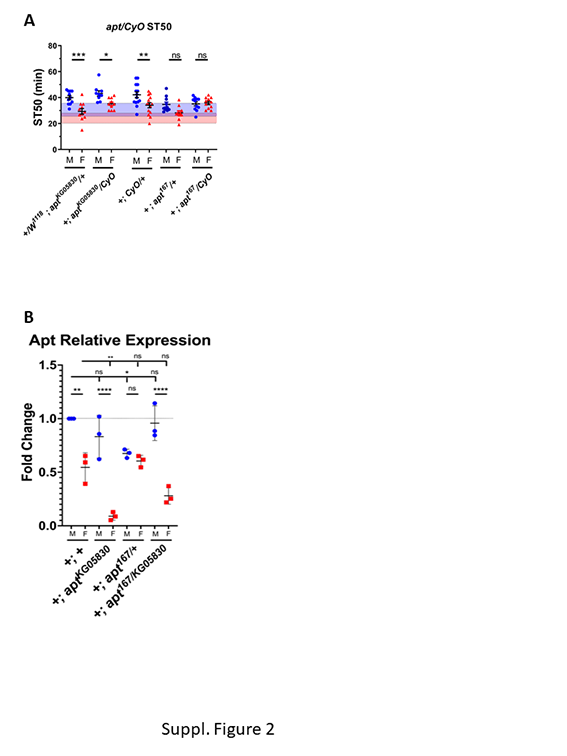

Supplement: Supplementary Figure 2 — (A) Heterozygous apt mutant flies show similar trends in sedation responses between males and females in different genetic backgrounds. Animals heterozygous for the aptKG05830 allele and either for the w1118 background or the CyO balancer show sexually dimorphic sedation responses similar to aptKG05830/+ flies heterozygous for the DGRP-774 background as shown in Figure 5C. For the apt167 allele, the progeny from the same cross (between females from the apt167/CyO stable line and DGRP-774 males) was compared. Whereas no significant dimorphic responses were observed in apt167/+ heterozygotes, dimorphism was observed between female and male CyO/+ siblings. Statistical significance was tested by one-way ANOVA with Šidák corrections and is shown by ***p < 0.001, **p < 0.01, and *p < 0.05. (B) apt and actin42A transcript levels were measured by qPCR from replicates using 3–7 days old flies. Fold change expression is normalized to DGRP-774 males. Significance values are shown for comparisons in-sex to DGRP-774 flies and between sexes within each genotype. [file Image_2.tif]
