# Supplementary material for: Corazonin Neurons Contribute to Dimorphic Ethanol Sedation Sensitivity in Drosophila melanogaster
Source: Front Neural Circuits. 2022 Jun 22;16:702901. doi: 10.3389/fncir.2022.702901 (PMC9256964; doi:10.3389/fncir.2022.702901)
Supplement: Supplementary Table 1 — Details of the statistical analyses conducted in this study. [file Data_Sheet_1.PDF]

Figure panel	Genotype	Mean Diff.	Statistical test	p- value
Fig. 2D	DGRP-774 M vs. DGRP-774 F	5.367	One-way ANOVA, Šídák's multiple comparisons test (MCT)	0.0015
	DGRP-73 M vs. DGRP-73 F	6.532	One-way ANOVA + Šídák's MCT	0.0074
	DGRP-229 M vs. DGRP-229 F	6.166	One-way ANOVA + Šídák's MCT	0.0136
	DGRP-304 M vs. DGRP-304 F	7.891	One-way ANOVA + Šídák's MCT	0.0006
	w^{1118} M vs. w^{1118} F	2.497	One-way ANOVA + Šídák's MCT	0.7406
	Oregon-R M vs. Oregon-R F	-3.531	One-way ANOVA + Šídák's MCT	0.4118
	Canton-S M vs. Canton-S F	1.039	One-way ANOVA + Šídák's MCT	0.9985
Fig. 3E	30% M vs. 30% F	6.733	One-way ANOVA + Šídák's MCT	0.0029
	50% M vs. 50% F	6.409	One-way ANOVA + Šídák's MCT	0.0051
	65% M vs. 65% F	5.993	One-way ANOVA + Šídák's MCT	0.0106
	73% M vs. 73% F	5.367	One-way ANOVA + Šídák's MCT	0.0005
	85% M vs. 85% F	0.8364	One-way ANOVA + Šídák's MCT	0.9984
	100% M vs. 100% F	0.8689	One-way ANOVA + Šídák's MCT	0.9980
Fig. 3F	50% M vs. 50% F	5.917	One-way ANOVA + Šídák's MCT	0.0035
	65% M vs. 65% F	3.526	One-way ANOVA + Šídák's MCT	0.0420
	73% M vs. 73% F	2.851	One-way ANOVA + Šídák's MCT	0.3905
	85% M vs. 85% F	0.4575	One-way ANOVA + Šídák's MCT	0.9996
	100% M vs. 100% F	0.5670	One-way ANOVA + Šídák's MCT	0.9988
Fig. 4C	UAS-Tra /+ M vs. UAS-Tra, Crz-GAL4 M	7.744	One-way ANOVA + Šídák's MCT	0.0074

	CrzGAL4/+ M vs.<br>UAS-Tra, crz-GAL4 M	10.42	One-way ANOVA +<br>Šídák's MCT	<0.0001
	UAS-Tra /+ F vs.<br>UAS-Tra, crz-GAL4 F	-3.176	One-way ANOVA +<br>Šídák's MCT	0.7800
	CrzGAL4/+ F vs.<br>UAS-Tra, crz-GAL4 F	1.553	One-way ANOVA +<br>Šídák's MCT	0.9962
	UAS-Tra /+ M vs.<br>UAS-Tra/+ F	6.792	One-way ANOVA +<br>Šídák's MCT	0.0455
	CrzGAL4/+ M vs.<br>CrzGAL4/+ F	4.735	One-way ANOVA +<br>Šídák's MCT	0.2223
	UAS-Tra, crz-GAL4 M vs.<br>UAS-Tra, crz-GAL4 F	-4.128	One-way ANOVA +<br>Šídák's MCT	0.3638
Fig. 5C	DGRP-774 M vs.<br>DGRP-774 F	5.993	One-way ANOVA +<br>Šídák's MCT	0.0106
	+; apt$^{KG/+}$ M vs.<br>+; apt$^{KG/+}$ F	6.517	One-way ANOVA +<br>Šídák's MCT	0.0230
	+; apt^{KG05830} M vs.<br>+; apt^{KG05830} F	4.776	One-way ANOVA +<br>Šídák's MCT	0.2030
	+; apt$^{167/+}$ M vs.<br>+; apt$^{167/+}$ F	6.500	One-way ANOVA +<br>Šídák's MCT	0.0525
	+; apt$^{167/KG05830}$ M vs.<br>+; apt$^{167/KG05830}$ F	4.752	One-way ANOVA +<br>Šídák's MCT	0.2273
	774 M vs. +; apt$^{KG/+}$ M	-5.731	One-way ANOVA,<br>Holm-Šídák's MCT	0.0773
	774 M vs. +; apt^{KG05830} M	-4.531	One-way ANOVA,<br>Holm-Šídák's MCT	0.1074
	774 M vs. +; apt$^{167/+}$ M	-6.542	One-way ANOVA,<br>Holm-Šídák's MCT	0.0730
	774 M vs.<br>+; apt$^{167/KG05830}$ M	-8.092	One-way ANOVA,<br>Holm-Šídák's MCT	0.0205
	774 F vs. +; apt$^{KG/+}$ F	-5.205	One-way ANOVA,<br>Holm-Šídák's MCT	0.0351
	774 F vs. +; apt^{KG05830} F	-5.747	One-way ANOVA,<br>Holm-Šídák's MCT	0.0351
	774 F vs. +; apt$^{167/+}$ F	-6.033	One-way ANOVA,<br>Holm-Šídák's MCT	0.0351
	774 F vs.<br>+; apt$^{167/KG05830}$ F	-9.331	One-way ANOVA,<br>Holm-Šídák's MCT	0.0007
Fig. 5D	UAS-apt-RNAi M vs.<br>UAS-apt-RNAi F	11.01	One-way ANOVA +<br>Šídák's MCT	0.0001
	Crz-GAL4/+ M vs.<br>Crz-GAL4/+ F	1.437	One-way ANOVA +<br>Šídák's MCT	0.9628

	Crz-GAL4/UAS-apt-RNAi M vs. Crz-GAL4/UAS-apt-RNAi F	5.840	One-way ANOVA + Šídák's MCT	0.0437
Suppl. Fig. 2	+/w^{1118} ; apt$^{KG/+}$ M vs. +/w^{1118} ; apt$^{KG/+}$ F	10.60	One-way ANOVA + Šídák's MCT	0.0009
	+; apt^{KG}/CyO M vs. +; apt^{KG}/CyO M F	8.013	One-way ANOVA + Šídák's MCT	0.0385
	+; CyO/+ M vs. +; CyO/+ F	7.994	One-way ANOVA + Šídák's MCT	0.0067
	+; apt$^{167/+}$ M vs. +; apt$^{167/+}$ F	6.500	One-way ANOVA + Šídák's MCT	0.1238
	+; apt^{167}/CyO M vs. +; apt^{167}/CyO F	-0.6818	One-way ANOVA + Šídák's MCT	0.9997
